# The Effects of Dietary Protein Supplementation on Exercise-Induced Inflammation and Oxidative Stress: A Systematic Review of Human Trials

**DOI:** 10.3390/antiox11010013

**Published:** 2021-12-22

**Authors:** Abrar Alhebshi, Nehal Alsharif, Josh Thorley, Lewis J. James, Tom Clifford

**Affiliations:** 1 School of Sport, Exercise and Health Sciences, Loughborough University, Loughborough LE11 3 TU, UK; a.alhebshi@lboro.ac.uk (A.A.); n.alsharif@lboro.ac.uk (N.A.); j.thorley@lboro.ac.uk (J.T.); l.james@lboro.ac.uk (L.J.J.); 2 Department of Clinical Nutrition, Faculty of Applied Medical Sciences, Umm Al-Qura University, Makkah 24382, Saudi Arabia; 3 Department of Clinical Nutrition, Faculty of Applied Medical Sciences, King Abdulaziz University, Jeddah 21589, Saudi Arabia

**Keywords:** oxidative damage, immunity, amino acids, high intensity exercise, exercise recovery

## Abstract

This systematic review examined the effects of whole protein and commonly consumed amino acid supplements on markers of exercise-induced inflammation and oxidative stress and was reported according to the PRISMA guidelines. MEDLINE and SPORTDiscus were searched from inception until June 2021. The inclusion criteria were randomized clinical trials in humans, healthy adult participants (≥18 years), dietary protein/amino acid interventions, and measurements of oxidative stress/the redox status or inflammation post-exercise. The Cochrane Collaboration risk of bias 2 tool was used to critically appraise the studies. Data extracted from thirty-four studies were included in the systematic review (totaling 757 participants with only 10 females; age range 19–40 years). The included trials examined five types of whole protein and seven different amino acids supplements; most studies (*n* = 20) failed to identify statistically significant effects on markers of inflammation or oxidative stress after exercise; some (*n* = 14) showed either anti-inflammatory or antioxidant effects on some, but not all, markers. In conclusion, we found weak and inconsistent evidence that dietary protein/amino acid interventions can modify exercise-induced changes in oxidative stress and inflammation. However, given that these were not the primary outcomes in many of the included studies and many had design limitations, further research is warranted (Open Science Framework registration number: 10.17605/OSF.IO/AGUR2).

## 1. Introduction

High-intensity, unaccustomed, or prolonged exercise imposes a high physiological demand that perturbs homeostasis and may result in skeletal muscle damage [1]. The precise mechanisms of exercise-induced muscle damage (EIMD) are yet to be established, but it likely stems from a combination of mechanical and metabolic stress [2,3]. Classical signs of EIMD include muscle soreness, limb swelling, loss of muscle function, and increased release of cytoskeletal proteins such as creatine kinase and myoglobin [1,4]. In addition, EIMD is associated with an acute inflammatory response, characterized by increases in circulatory and intramuscular inflammatory markers such as C-reactive protein (CRP), cytokines (tumor necrosis factor (TNF-α) and interleukin-6 (IL-6)), and transcription factor nuclear factor kappa beta (NF-κB) [5,6,7]. Exercise also increases the production of reactive oxygen (ROS) or reactive nitrogen (RNS) species [8,9], which partly stem from immune cells such as neutrophils [10]. Nonetheless, muscle damage is not necessary for an inflammatory response after exercise; indeed, inflammation and increased generation of ROS/RNS (herein referred to as RONS) can occur without overt signs of muscle damage [11], often after high-intensity or prolonged aerobic exercise [5,12,13].

The inflammatory response and increase in RONS after exercise are necessary and undoubtedly important for both adaptative and regenerative responses [3,14]. However, an excessive response may have negative consequences; for example, an increase in RONS that overwhelms the endogenous antioxidant system can damage lipids, proteins, and DNA, leading to cell dysfunction [15,16]. It has also been postulated that, in the process of repairing tissues, phagocytes may injure neighboring healthy components of the cell [17] and that an excessive rise in RONS may impair muscle contractions and force production [18,19]. In the context of sports and exercise, the inflammatory response may inhibit the recovery of muscle function and/or sustain muscle soreness, impeding an athlete’s short-term recovery [11,20,21]. However, more importantly, in many chronic disease states, RONS and inflammation are elevated, propagating dysfunction and worsening patient outcomes [22,23]. Thus, an active area of research is determining what interventions might modify the inflammatory response and/or RONS production to enhance exercise recovery/rehabilitation and/or improve patient health. As high-intensity exercise evokes a robust inflammatory response and an increase in many markers of oxidative stress (an imbalance between oxidants and antioxidants) that may lead to oxidative distress (impaired redox signaling and/or damage to biomolecules [24]), it is considered a useful model to study the effectiveness of interventions purported to modify these responses [14,25].

After or between strenuous exercise bouts, athletes and recreational exercisers may use a variety of different nutritional strategies to enhance recovery and minimize inflammation/RONS or other EIMD symptoms [26]. Protein-based supplements, especially protein powders such as whey, soy, and casein, are widely consumed and have been shown to increase muscle protein synthesis and accelerate skeletal muscle remodeling following exercise [27,28,29]. However, the impact of dietary proteins on the acute inflammatory and RONS responses to exercise is rather limited.

Some studies report that whey protein has antioxidant effects, attributed to its ability to enhance the availability of glutathione, a cellular thiol, and increase the endogenous antioxidant enzymes superoxide dismutase, glutathione peroxidase, and catalase [30,31]. Evidence in animals [32,33,34] and cell lines [35,36,37] has shown that whey protein modifies several intracellular pathways related to RONS production, and this, in turn, can support normal immune responses to trauma. Soy protein, either in its isolated form or isoflavone-enriched, may attenuate chronic inflammation by dampening NF-κB signaling and the secretion of proinflammatory cytokines (TNF-α, IL-6, and interleukin-1-beta (IL-1β)) [38,39]. There are also suggestions that popular supplements formed of one or more amino acids, such as branch-chain amino acids (BCAAs), L-glutamine, or glycine, have anti-inflammatory effects [40,41]. Glutamine can modify cytokine release, possibly by increasing heat shock protein 72 expression [42] and/or attenuating NF-κB activation [43]. BCAAs anti-inflammatory effects are purportedly indirect, a consequence of increased glutamine synthesis, or increased mammalian target of rapamycin (mTOR) activation, which subsequently decreases proteolysis [44]. Glycine, the major component of collagen peptides, has been shown to blunt superoxide production by neutrophils via a calcium-dependent pathway [45]. The amino acid L-carnitine has been shown to attenuate exercise-induced oxidative stress in humans, ostensibly indirectly, by enhancing muscle oxygenation during exercise and thereby limiting the activation of calcium-dependent proteases and xanthine oxidase, which generates superoxide [46,47]. Yet, despite the strong mechanistic support from in vitro studies, the evidence of immunomodulatory or antioxidant effects in human trials is mixed. Although several studies [8,30,48,49,50] have reported that protein supplementation might, to some extent, accelerate muscle function recovery by mitigating aberrant inflammation or oxidative stress post-exercise, others found negligible effects [51,52,53,54,55,56]. 

The equivocal findings to date are, at least partly, due to the differences in study designs, such as types of exercise, outcome measures, training status, and types of supplements and their doses. Therefore, the aim of this article is to systematically review the current evidence from high-quality randomized controlled trails examining the effect of whole protein supplements (e.g., whey, casein, and soy) or high-protein foods on markers of exercise-induced inflammation and oxidative stress. A secondary aim is to examine the effects of commonly consumed amino acid supplements purported to modify inflammation/RONS after exercise (e.g., BCAA, L-glutamine, and collagen peptides).

## 2. Materials and Methods

This systematic review was preregistered on the Open Science Framework database (registration number: 10.17605/OSF.IO/AGUR2) and was conducted and reported according to the recommendations of the Preferred Reporting Elements for Systematic reviews and Meta-analysis (PRISMA) guidelines [57].

### 2.1. Search Strategy 

**Figure 1:**
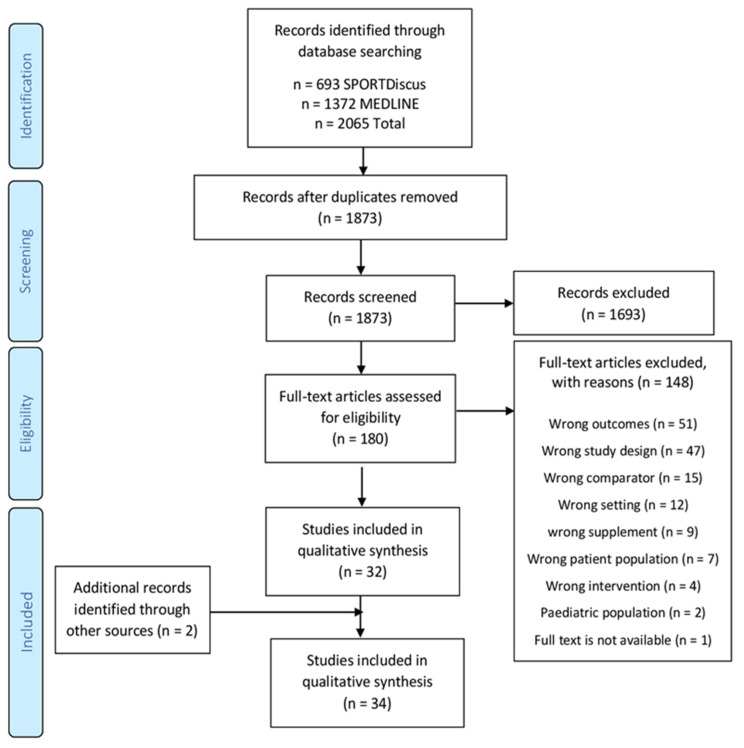
PRISMA flow diagram of the literature search strategy.

The databases MEDLINE and SPORTDiscus were searched from inception until June 2021 to identify relevant studies that were limited to English full-text articles only. Our search strategy was developed in accordance with a population, intervention, comparator, outcome, and study design (PICOS) methodology. Our full search strategy is available in the online Supplementary Materials. The PICOS strategy was designed using previous systematic review articles with similar outcomes [58,59]. The search results were exported to Covidence software (Covidence, Melbourne, VIC, Australia) for duplicate removal and screening. The title and abstract of the retrieved articles were independently screened by two authors (A.A. and J.T.). Using the predefined eligibility criteria outlined below, the relevant articles’ full texts were assessed for eligibility by both investigators independently. A manual search of all the included full-text articles was performed for any additional studies. The final studies were included upon the agreement of both authors; however, some conflicts were resolved by the senior author (T.C). Figure 1 shows the results of our search strategy.

### 2.2. Study Selection 

The eligibility screening of the relevant articles and data extraction was performed according to specific criteria, which are delineated below:

The inclusion criteria were: (1) randomized clinical trials conducted in human subjects. No distinction was made for the specific designs of the trials (duration, placebo-controlled, crossover, or blinding); (2) adult participants (age ≥ 18 years); and (3) studies based on protein supplementation were included if they provided information on the type of protein (whey, casein, or soy); dose; formulation; frequency; and route of administration. Studies based on protein food ingestion were included in the systematic review if they provided information on the frequency and amount of food provided. Only studies in which the effects of protein can be isolated from other nonprotein nutrients were eligible. Supplements or protein foods that were not included in our search terms but met the inclusion criteria were still deemed eligible. (4) Markers of oxidative stress/redox status or inflammation at any timepoint <96 h following acute bouts of exercise in any tissue (e.g., circulation and muscles). Previous literature was used to determine whether a marker represented these processes [3,60]. (5) Full-text papers and abstracts were eligible (abstracts were only eligible if they contained sufficient information to complete qualitative and quantitative analyses). 

Studies were excluded if any of the following criteria were present: (1) any type of longitudinal observational studies and nonrandomized clinical interventions, training studies or studies with multiple bouts of exercise performed alongside supplementation, and studies performed in extreme environmental conditions (e.g., altitude); (2) studies including nonadult subjects and/or patient populations; and (3) if the protein supplement was consumed alongside a substantial amount of a nonprotein intervention purported to modulate inflammation (e.g., (poly)phenols, vitamin C, creatine, or carbohydrates) and an appropriate (well-matched) control group/placebo was not evident. If a negligible amount of the nonprotein intervention was present (e.g., carbohydrates < 10 g) and an appropriate control group was present, then this was not excluded. (4) Studies were also excluded if the outcome markers were only measured pre-exercise or not after an exercise bout. If we were unable to obtain any information requested, we excluded the manuscript.

### 2.3. Data Extraction

Relevant data were extracted and tabulated into a Microsoft Excel spreadsheet by the first author (A.A.) and were validated independently by a second author (J.T.). Extracted data were based on our predefined PICOS (see the online Supplementary Materials) and included: Authors and publication year; study design and duration; participant characteristics (number, gender, and age); intervention and comparator characteristics (type, component, dose, and timing); exercise protocol (type, duration, repetitions, and sets); and relevant outcome measures at the baseline and various postintervention timepoints. Eligible outcomes were any inflammatory or oxidative stress markers taken < 96 h post-exercise. Extracted data are displayed in Table 1 and Table 2.

### 2.4. Quality Assessment

The quality of the included studies was assessed using the Cochrane Collaborations Risk of Bias 2 tool [61]. Parallel and crossover studies were assessed separately using predesigned spreadsheets available at https://www.riskofbias.info/ (accessed on 5 October 2021). The assessments were completed independently by two authors (A.A. and N.A.), and discrepancies were resolved through discussion and the judgement of a third author (T.C.). The quality of each study was classified as either a low risk of bias, some concerns, or a high risk of bias, according to the following domains: (1) allocation sequence randomization and concealment, (2) blinding, (3) missing outcome data, (4) outcome measurement bias, (5) selective reporting, and other crossover trial-related bias arising from duration and carryover effects.

## 3. Results

### 3.1. Search Results

The results from our search strategy are displayed in Figure 1. A total of 2065 articles were retrieved from two databases. After eliminating duplicates, 1873 studies remained. After title and abstract reviews, 1693 were excluded; 180 studies remained for the full-text assessment; of these, 148 were excluded for various reasons (see Figure 1), with 32 deemed eligible for the systematic review. Two further studies were identified through the manual search of the reference lists of the included articles [48,62]. In total, 34 studies met our eligibility criteria for inclusion in the systematic review (Table 1 and Table 2). A meta-analysis was deemed inappropriate due to the wide heterogeneity of the eligible studies. 

### 3.2. Study Characteristics

Of the 34 included studies, 19 used a parallel group design [48,49,51,52,53,54,55,63,64,65,66,67,68,69,70,71,72,73,74], 3 had three treatment arms [52,54,63], and 2 had four treatment arms [68,74]. The remaining 15 studies employed a crossover design [8,30,50,56,75,76,77,78,79,80,81,82,83,84,85], three of which had three treatment arms [50,80,81].

Across all the studies, over fifty different markers were used to measure exercise-induced inflammation or oxidative stress (see Table 1 and Table 2). Eighteen trials investigated the effect of whole protein sources (whey, milk, soy, pea, and casein), while the other sixteen trials included various single or combined amino acids as one supplement. The length of times the interventions were provided for ranged from a single dose to 4 weeks (see Table 1 and Table 2). 

The number of participants in the review was 757; only 1.3% (10 participants) were reported as female. The sample sizes were relatively small, with an average size of 15, ranging from 8–40. The mean age of all the participants was 24 years, with an age range of 19–40 years. Nineteen studies recruited healthy athletes or highly trained professionals [8,30,48,50,55,56,65,70,71,74,76,77,78,79,80,82,83,84,85], and nine studies were conducted on recreationally active participants or participants that ﻿habitually performed resistance exercise training 1 to 2 days a week [51,53,54,66,67,69,72,73,81]. Six studies were on untrained participants [49,52,63,64,68,75].

### 3.3. Whole Protein Interventions 

Milk. Four studies investigated the effect of milk consumption following muscle-damaging exercise. One study showed that a milk protein supplement (80 g/day) attenuated protein carbonyls (PC), a marker of protein oxidation, 1 day (*p* = 0.002), 2 days (*p* = 0.034), and 8 days (*p* = 0.037) post-exercise compared to a placebo group [8]. No differences (*p* > 0.05) were found for the leukocyte counts, NF-κB phosphorylation, and 70 kilodalton heat shock proteins (HSP70) between the placebo and milk groups. By contrast, Rankin et al. (2017) found that 500 mL of milk following eccentric exercise had no impact on the PC, high-sensitivity CRP (hsCRP) or reduced glutathione-to-oxidized glutathione ratio (GSH/GSSG). Similarly, three other studies found that milk-based beverages had no influence on several inflammatory markers post-exercise, including: TNF-α, tumor necrosis factor receptor 1 (TNFr1) expression on monocytes (*p* > 0.05) [85], plasma monocyte chemoattractant protein 1 (MCP-1) (*p* = 0.48), chemokine receptor type 2 (CCR2) expression (monocyte chemotaxis) (*p* = 0.23), CD11 b expression (monocyte adhesion) (*p* = 0.12), proportion of CD14+ relative to all leukocytes (*p* = 0.27), CD14++CD16- monocytes relative to all monocytes (*p* = 0.61) [84], IL-6, interleukin-1, and TNF-α (*p* > 0.05) [52].

Casein Protein. One trial examined the impact of consuming a carbohydrate-rich casein and leucine supplement every 15 min during cycling exercise and reported a post-exercise reduction in circulating neutrophils (*p* = 0.034) and lymphocytes (*p* = 0.048) [79]. 

Whey protein. Of the twelve studies with whey protein supplementation, only two demonstrated favorable anti-inflammatory or antioxidant effects [30,50], with the remaining ten reporting no effects [51,54,63,66,75,76,78,80,81,82]. 

Whey protein concentrate. In a study that showed anti-inflammatory effects, a carbohydrate–whey protein cake consumed after 2 h of exhaustive cycling exercise reduced IL-6 (−50%) and CRP (−46%) at 4 h post-exercise (*p* < 0.05) [30]. 

Twice-daily intake of a whey protein–carbohydrate drink before and after eccentric exercise did not suppress post-exercise changes in CRP (*p* = 0.57) [51].

Similarly, in Grubic et al. [78], a whey protein-enriched bar (20-g protein) consumed before, during, and after resistance exercise had no effect on various cytokines: interferon gamma (IFNy) (*p* = 0.56), interleukin 13 (IL-13) (*p* = 0.563), IL-1β (*p* = 0.47), interleukin 4 (IL-4) (*p* = 0.37), IL-6 (*p* = 0.43), interleukin 8 (IL-8) (*p* = 0.57), and TNF-α (*p* = 0.261). 

Whey protein isolate. Seven studies examined the effect of whey protein isolate on markers of exercise-induced inflammation and oxidative stress; none of them reported changes with the intervention [54,63,66,75,76,80,82]. In Baba et al. [75], whey protein isolate (22.8 g) before, during, and after exercise did not attenuate IL-6 4 h post-exercise (*p* = 0.055). In another crossover trial, the ingestion of a whey protein isolate–carbohydrate drink during and following 90 min of intermittent running had no influence on any systemic indices of inflammation (IL-6, interleukin 10 (IL-10), CRP, and IL-1 receptor antagonist) compared to a carbohydrate-only drink [76]. Supplementation before and after eccentric exercise with 25 g of whey protein isolate or hydrolysate had no effect on the plasma TNF-α concentrations (*p* = 0.93) [63]. Likewise, Karakuş and Akkurt [66] found no effect of whey protein after resistance training on circulating neutrophils, platelets, leukocytes, and lymphocytes (*p* > 0.05). In Kritikos et al. [80], soccer players who consumed whey protein in doses to reach 1.5 g/kg/day had no effect on the markers of blood redox status (*p* > 0.05; see Table 1 for specific markers). Consuming whey protein (3× daily 0.3 g/kg) before and after eccentric exercise had no effect on CRP (*p* = 0.133) [54]. In Rothschild et al. [82], a pre-exercise whey protein-enriched meal did not attenuate the F_2_-isoprostane levels (*p* = 0.595).

Whey protein and amino acid combinations. In a comprehensive study, Rowlands et al. [50] found that the ingestion of a leucine-enhanced whey drink following high-intensity cycling exercise activated pro-regenerative inflammatory transcriptome networks < 240 min post-exercise compared to an isocaloric control beverage. Specifically, muscle leukocyte maturation (*p* = 0.007), cell cycle arrest (*p* = 0.01), cell viability (*p* = 0.005), IL-1 I-centered proinflammatory network and leukocyte migration, differentiation, and survival (*p* < 0.001) were enhanced with the intervention. Another study found no impact of an L-glutamine- and L-carnitine-L-tartrate-enriched carbohydrate–protein supplement compared to a carbohydrate or a low-calorie placebo on IL-6 after 90 min of intermittent exercise (*p* = 0.196) [81].

Plant protein. Kritikos et al. [80] found that soy protein isolate intake in a dose of 1.5 g/kg/day reduced PC 48 h following a speed–endurance exercise (*p* = 0.04). A 4-week intervention of soy protein isolate (21-g soy) twice daily in forty male boxers and cyclists had no effect on the hsCRP and myeloperoxidase (MPO) following 100 drop jumps [70]. Nieman et al. [54] found no effect of pea protein (3× daily 0.3 g/kg) on CRP after eccentric exercise (*p* = 0.133).

### 3.4. Amino Acid Interventions

Amino acid combinations. Five studies investigated the effect of combining different amino acids on biomarkers of oxidative stress and inflammation after exercise. One reported anti-inflammatory effects [68], one equivocal effect [73], and the remaining three showed no effect [65,72,84]. Ra et al. [68] found that a combined taurine and BCAA supplement consumed for two weeks prior to and after eccentric elbow flexor exercise significantly lowered the serum levels of 8-hydroxydeoxyguanosine (8-OHdG), a marker of DNA damage, compared to the placebo and BCAA and placebo-only groups (*p* < 0.05) but not compared to a taurine plus placebo group. Waskiw-Ford et al. [73] found that the muscle heat shock protein 72 (HSP72) levels decreased significantly (*p* = 0.038) following leucine-enriched essential amino acid ingestion thrice daily for four days after an acute bout of lower-body resistance exercise, with no significant changes in the heat shock protein 25 (HSP25) (*p* = 0.66) and IL-6 (*p* = 0.13) concentrations during recovery compared with a carbohydrate placebo. Similarly, Jackman et al. [65] found no effect of BCAAs (four doses pre- and post-eccentric knee extension exercise) on the systemic IL-6 levels (*p* > 0.05). In another study, a leucine-enriched essential amino acid drink before and after resistance exercise had no effect on the mRNA expression of IL-6 and IL-1β (*p* > 0.05) [72].

β-hydroxy-β-methyl butyric acid. β-hydroxy-β-methylbutyrate before and after a high-volume full-body resistance training session had no effect on the plasma CRP level 48 h post-exercise (*p* = 0.29) [55].

L-carnitine. Two studies investigated the influence of L-carnitine on oxidative stress markers following exercise [49,83]. Daily supplementation with 2 g of L-carnitine for 2 weeks before a 14-km run increased the total antioxidant capacity (TAC) (*p* = 0.02) and attenuated malondialdehyde (MDA) and thiobarbituric acid-reactive substances (TBARS) (*p* = 0.003) 24 h post-exercise [49]. Volek et al. [83] reported that 2 g/day of L-carnitine L-tartrate consumption for 3 weeks before back squat training and throughout recovery significantly lowered the plasma MDA and xanthine oxidase (XO) concentrations at selected timepoints post-exercise (*p* ≤ 0.05).

L-citrulline. Six grams of L-citrulline-malate supplementation 2 h before cycling exercise significantly increased ROS production by the polymorphonuclear neutrophil (PMN) concentration only in the supplemented group after exercise (*p* < 0.05); there were no significant changes in MDA and DNA damage in both the supplement and control (lemon juice) groups [71].

Collagen peptides. Clifford et al. [53] found no effect of collagen peptides (CP) (20 g/day for 9 days pre- and post-muscle-damaging exercise) on any blood inflammatory markers: leukocytes, neutrophils, monocytes, lymphocytes, and IL-6 (*p* > 0.05). 

Glutamine. Three studies examined the impact of glutamine ingestion on inflammation and oxidative stress markers after exercise; two reported no effects [48,77], and one reported favorable effects [67]. Cury-Boaventura et al. [77] found that supplementation with 50 g of maltodextrin plus 2.8-g hydrolyzed whey protein enriched with 175 mg of glutamine thirty minutes before exhaustive running had no effect on ROS production by neutrophils and DNA fragmentation in neutrophils and lymphocytes compared to a control (*p* > 0.05) who consumed 50 g of maltodextrin alone. In another study, consuming 1.5 g/kg of glutamine for 7 days prior to running did not affect the post-exercise changes in TAC, glutathione, or MDA (*p* > 0.05) [48]. In contrast, Nemati et al. [67] found that 0.3 g/kg of glutamine for 2 weeks prior to exhaustive exercise significantly increased the systemic TAC (*p* = 0.01) and glutathione (*p* = 0.002) and reduced the post-exercise levels of serum MDA (*p* = 0.04) and hsCRP (*p* = 0.02). 

Taurine. Three studies evaluated the effects of taurine [64,69,74]. A study comparing 50 mg/kg of taurine and starch for 21 days pre- and post-eccentric exercise observed a significant reduction in the xylenol levels in the 7 days post-exercise (*p* < 0.05). The PC were significantly lower in the taurine group post-exercise (*p* < 0.05), and the total protein thiol (TT) content was significantly higher (*p* < 0.05) post-exercise. No significant changes were detected for the erythrocyte activities of superoxide dismutase (SOD), catalase (CAT), glutathione peroxidase (GPX), TNF-α, IL-1β, and IL-10 post-exercise (*p* > 0.05) [64]. In another study, 6 g/day of taurine for 18 days significantly decreased the serum MDA concentration (*p* < 0.05) 3 and 4 days after eccentric muscle contractions [69]. One large study evaluated the effectiveness of 3 g/day of taurine vs. 0.35-g/day saccharum lactis placebo in three doses for 3 days before an intense full-body resistance exercise. SOD and GPX activity were significantly higher in the taurine treatment group at 24 h post-exercise compared to the placebo (*p* ≤ 0.0001 and *p* = 0.033, respectively). The taurine supplement had no significant effect on the TBARS, plasma protein thiols, and CAT activity at any timepoint after exercise (*p* > 0.05) [74].

### 3.5. Risk of Bias 

**Figure 2:**
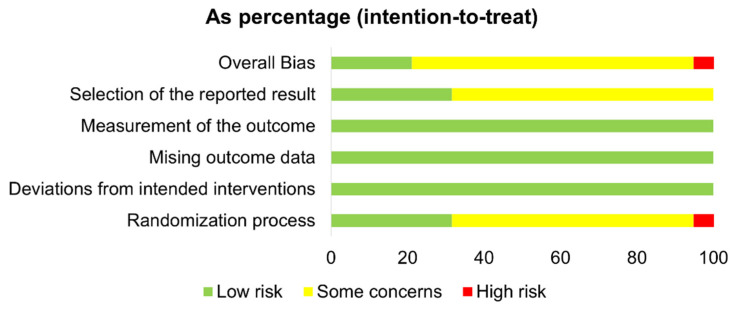
Risk of bias graph for the parallel design trials.

**Figure 3:**
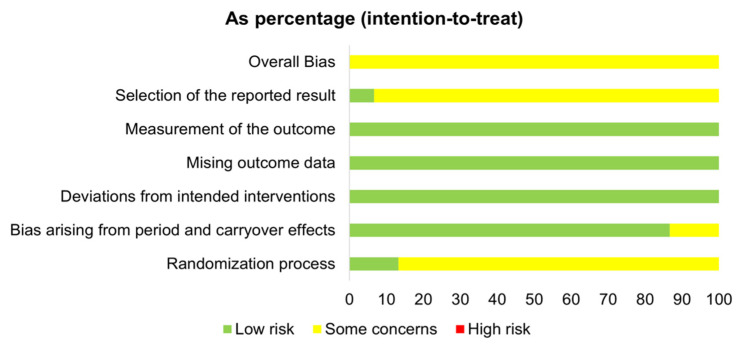
Risk of bias graph for the crossover trials.

The risk of bias summaries for all the trials are presented in Figure 2 and Figure 3 and Figures S2 and S3 in the online Supplementary Materials Figures (individual summaries). In the parallel design trials, *n* = 14/19 were rated to have some concerns (Figure 2). Most were due to inadequate descriptions of their randomization and allocation concealment procedures (*n* = 12). Most studies (*n* = 12) did not have a prespecified analysis plan. Four [51,54,72,73] of the trials had a low risk of bias for all the variables; none of these noted a significant impact of protein intervention on inflammatory markers after exercise. One study [66] was given a high risk of bias, because they did not report any information about the allocation sequence randomization and concealment, and the distribution of participants between the treatment groups was imbalanced: 15 participants in the intervention group and nine participants in the control group. 

For the crossover trials, all 15 were rated to have some concerns overall (Figure 3). Most of the studies (12/15) were rated to have some concerns, because insufficient information was given on the randomization and allocation concealment procedures. Two had some concerns for the period and carryover effects domain [50,82]. The risk of bias arising from the selection of the reported results had some concerns in all except one trial [78], as no information about a prespecified analysis plan was available.

## 4. Discussion

Dietary protein interventions have been suggested to modify inflammation and redox status and may therefore support recovery from high-intensity and/or muscle damaging exercise or help to manage low-grade chronic inflammation associated with aging and noncommunicable diseases. This is the first systematic review evaluating the effects of various whole protein and amino acid supplements and foods on exercise-induced inflammation and oxidative stress in humans. The 34 included studies examined five types of whole protein and seven different amino acids supplements, of which milk, whey protein, and mixed amino acids drinks were the most frequent. Most studies (*n* = 20) failed to identify statistically significant effects on markers of inflammation or oxidative stress after exercise, whereas five studies on whole proteins [8,30,50,79,80] and nine studies on amino acids supplements [49,64,67,68,69,71,73,74,83] showed either anti-inflammatory or antioxidant effects on some markers when compared with the controls. Of all the protein interventions included in the review, L-carnitine was the only intervention to show consistent benefits; however, only two studies were included, and therefore, the level of evidence is weak. Overall, the findings were equivocal for all the protein interventions, with no consistent patterns emerging to confidently recommend a protein or amino acid intervention and dosage that significantly alters the post-exercise inflammation or redox status in healthy young individuals. The inconsistent findings are likely due to the wide heterogeneity of the study designs, the poor control of the interventions and diet (in some studies), the limited range of the biomarkers measured, and lack of statistical power to detect significant effects.

To our knowledge, no other reviews have examined the effect of protein interventions on exercise-induced inflammation and oxidative stress, but there are reviews examining the effect of proteins on surrogate markers of muscle damage and recovery after exercise. Our results are in line with Pasiakos et al. [86], who, in their systematic review of 27 studies, concluded that there was limited evidence that whole protein interventions can attenuate markers of muscle damage such as a loss of force-generating capacity and muscle soreness. By contrast, in a meta-analysis of 13 trials, Davies et al. [87] found whey protein supplementation to have a small-to-medium effect on the recovery of muscle function in the 24–96 h after exercise. They did not include markers of inflammation or oxidative stress for a more direct comparison to our findings, but they did measure the changes in creatine kinase, a marker of muscle damage, and found no benefit of whey protein. Our findings also differed with a large systematic review of 52 trials, which concluded that a protein-rich dairy intake has anti-inflammatory effects in humans [88]. However, they did not restrict their studies to RCTs as we did, and most of the included studies were of patients as opposed to young healthy adults. In another recent SR, this time with 16 RCTs and again with dairy products, long-term intake was shown to have a weak anti-inflammatory effect, and the data from acute studies were inconsistent and limited [89]. Taken together with our findings, there appears to be limited high-quality evidence that protein-based interventions can modulate acute inflammation and/or oxidative stress in humans.

Only one study was considered to have a high risk of bias, with most rated to have some concerns. Akin to several previous systematic reviews with nutritional interventions [58,90], most of the included studies were deemed to have some concerns, because there was insufficient information on the randomization and allocation concealment procedures, as well as missing information about a prespecified analysis plan in each study. The former is potentially problematic because it can result in selection bias [91] and the latter because, without evidence of a prespecified analysis plan, it is difficult to know whether any markers have not been reported or if the analysis was selected post hoc to fit the hypothesis [92].

A limitation of many studies was insufficient statistical power. Seventeen studies out of thirty-four included less than twenty participants, and only 11 studies conducted a power analysis based on the published results to justify their sample sizes [8,51,54,70,73,76,78,79,80,84,85]. In one study [8], the desired sample size was not reached, and therefore, the statistical power may have been compromised. The issue of small sample sizes or insufficient statistical power has been discussed at length previously [93], and in the many studies with low statistical power, the findings could have been biased, with the magnitude of the effect more likely to be exaggerated [94]. It is important to note that none of the studies completed the power calculations for all the reported oxidative stress and inflammatory markers, and of those that did, only one stated that it was for the primary outcome in the study [80]. Therefore, it is most likely that many of the studies were underpowered to detect any significant effects, and type two errors may have been prevalent.

Another possible source of bias in the studies was insufficient dietary control. Only two trials provided a prescribed nutritional plan for participants with [8] and without dietary records [66]. Many studies reported that the participants were advised to maintain their habitual diet and record their dietary intake, some with a food diary [30,51,53,56,64,67,78,80,81,82,84,85] and others without [49,68,69,77,79]. Seven studies provided no indication that the dietary intake was controlled or recorded [48,54,63,70,72,74,83], which would make it difficult to determine if the effects were due to differences in the dietary intakes or the interventions. Of those that reported controlling diets throughout the testing period with food provision [50,52,55,65,71,73,75,76], only three of those found that the protein supplementation had an anti-inflammatory effect [50,71,73]. Ideally, future studies would provide participants a specific diet to avoid variations in between trial dietary intakes. However, this can be expensive and logistically challenging, so, at the least, dietary intake should be recorded, and diaries scrutinized for any between trial differences in protein intakes and foods that could affect inflammation or oxidative stress, such as vitamin C or omega-3 fatty acids.

Regarding study design, several trials adopted a repeated-measures (*n* = 15) rather than a between-group (*n* = 19) experimental design. Although the repeated-measures studies often recruited highly trained cyclists or well-trained athletes and involved running or cycling (*n* = 10), those with resistance exercise protocols (*n* = 5) could have been influenced by the repeated bout effect, in which the extent of muscle damage, inflammation, and oxidative stress is lessened when the same stimulus is repeated several weeks later [1]. In addition, many studies (*n* = 6) were performed in participants described as untrained or unaccustomed to resistance exercise, which results in more robust changes in redox status and immune function, perhaps making it easier to detect significant effects if they are present. In untrained participants, a previous review suggested that protein supplements mostly, but not always, decreased surrogate markers of muscle damage, such as loss of the force-generating capacity [86]. However, this was not evident in our review, as all trials except two [64,68] in untrained healthy participants did not report significant changes in the post-exercise inflammation and redox status.

There was a large difference in the duration of interventions in the included studies, with no particular pattern emerging to suggest there was an optimal length of intake to alter immune or redox status. Only six studies gave a single dose of protein [56,71,77,82,84,85], and only one reported a significant change [71]. In most trials (*n* = 15), the participants were given multiple doses before, during, and after exercise [30,50,52,55,63,65,66,72,73,75,76,78,79,80,81]. Of those with a short duration protocol, only five showed a significant effect of protein on some measured markers of inflammation or oxidative stress [30,50,73,79,80]. The rest of the trials (*n* = 13) administered supplements for a longer duration (>5 days to ≤4 weeks), with only eight reporting significant effects on some inflammatory and oxidative stress markers [8,49,64,67,68,69,74,83]. Clearly, the study designs, types of protein, duration of intakes coupled with the inconsistent findings, mean that no definitive conclusions on the optimal dose of protein intake to modify the immune and redox status can be made.

Although high-intensity/strenuous exercise evokes oxidative stress and inflammation, and this may contribute to muscle soreness and loss of muscle force-generating capacity [1,21], we acknowledge that these biological processes are also important for training adaptations. Indeed, some [95,96], but not all, studies [97] have shown that interventions aiming to blunt inflammation and/or oxidative stress can attenuate exercise adaptations such as mitochondrial biogenesis and/or muscle growth. In fact, a recent review paper suggests athletes may seek methods to increase the prooxidant status of muscle to optimize such physiological adaptations [98]. Thus, if an athlete is trying to maximize training adaptations, avoiding interventions purported to blunt these responses would be advisable. By contrast, when the recovery time between competitions is limited (e.g., <72 h) and adaptions are not the main goal, anti-inflammatory or antioxidant interventions may accelerate functional recovery. Thus, interventions with such biological effects should only be considered in the correct context. Somewhat paradoxically, unlike the prevailing theory that traditional anti-inflammatory or antioxidant supplements such as nonsteroidal anti-inflammatory drugs or vitamin C inhibit exercise adaptations, dietary protein interventions support mitochondrial biogenesis and skeletal muscle growth [99,100]. This suggests that if dietary protein or amino acids modulate the immune or redox system, the effects are complimentary or permissive to these adaptations or at least not harmful. Rowlands and colleagues [50] lent some support to this theory. They showed that dietary protein, with high leucine (5–15 g), evokes an immune-driven pro-resolving effect on muscles post-exercise, characterized by NF-κB inhibition, earlier leukocyte maturation, and greater presence of macrophages. This study clearly showed that dietary protein and leucine dose-dependently influenced immune-related repair mechanisms post-exercise, leading the authors to conclude that the immune-driven events likely complement muscle protein turnover and remodeling. Further research is needed to understand how dietary protein and amino acid interventions could modulate post-exercise inflammation and redox status and what impact, if any, such changes have for functions and adaptations.

It is important to acknowledge the limitations of this review. Firstly, the aims of most of the included studies were chiefly not to focus on changes in the inflammation or redox status but, instead, mostly on performance, muscle damage, and exercise recovery. This may partly explain why few markers of inflammation or oxidative stress were measured in most studies, with many measuring ≤two markers. Across all the studies, the overall number of measured markers was low, with eight studies assessing only one biomarker [51,54,55,63,65,68,75,81], and of those that assessed more markers (≥four, *n* = 10), only half of them found a significant effect of the intervention compared to the placebo [8,64,67,71,74]. As no one specific marker is sufficient to represent inflammation or oxidative stress, it is currently recommended that studies include a comprehensive array of biomarkers where possible [101,102]. As such, findings from studies where only one marker was assessed should be interpreted with caution. Another limitation is that many of the redox markers used, such as TBARS and TAC, are no longer considered valid markers of redox status [102]. This, combined with the fact that many of the redox markers were not necessarily measured in the cell of interest (e.g., muscle) but, instead, in serum or plasma—where the origin of oxidation products is difficult to verify—could partly explain why few studies have reported statistically significant effects [102,103]. Another limitation in some studies was not matching the carbohydrate dosage in the comparator and intervention groups. Carbohydrate intake may modulate post-exercise immune function and redox status [104,105], and therefore, any potential effects of the primary treatment could have been missed. Another limitation of the review is that, to increase the homogeneity of the included studies, only studies in healthy young adults free from disease were eligible. This means these findings may not extend to older adults or diseased populations in whom exercise may elicit greater changes in inflammation and redox status and may therefore benefit more from these interventions. This review also mostly includes studies that assessed markers of inflammation and oxidative stress in the circulation (*n* = 29). Only five trials measured these markers in other cells, including muscles [8,50,72,73] and urine [82], and therefore, these conclusions are largely based on systemic changes. Nonetheless, only 50% of those measuring changes in muscles reported a significant change in some inflammatory markers [50,73], so it is unclear if different conclusions would be drawn if more studies were conducted in other tissues. Lastly, almost all the participants in this review were male (771 out of 781 participants), meaning the findings have limited generalizability for females. Another inevitable limitation of our review was the quality of the included studies. In addition to the generally low sample sizes, poor dietary control, and lack of clarity regarding the primary outcomes, most of the studies had some concerns in their risk of bias assessments.

## 5. Future Research

As no biomarker is known to identify inflammation/oxidative stress or to effectively determine the effectiveness of food supplements [59], future studies should aim to include a battery of markers that represents as much of the immune and redox signaling cascade as feasible. As an example, this could mean including pro- and anti-inflammatory markers alongside molecular signatures in various tissues. In addition, future studies should consider including multiple timepoints post-exercise, especially in the first few hours when inflammation is typically greater, to avoid missing any transient changes. Studies are also needed on females, as most data is currently restricted to males. Future studies should also explore individual responses to these interventions—specifically, whether the impact of dietary protein or amino acid interventions is at least partly dependent on the baseline redox/immune status. A recent study [106] showed that a high dietary cysteine intake corelated with higher blood glutathione levels, implying that the dietary interventions purported to modify redox status may have greater efficacy in deficient individuals. The effectiveness of this more personalized approached to nutrition interventions should be a focus of future research. Lastly, trials should ensure they have adequate dietary control, statistical power to detect an effect if present, and preregister their analysis plan to avoid selective reporting, which is especially important when several biomarkers are measured.

## 6. Conclusions

In conclusion, due to the inconsistent findings and potential bias in many studies, there is currently insufficient evidence that dietary protein or amino acid interventions could modify exercise-induced changes in oxidative stress and inflammation. The inconsistent findings and inability to draw more concrete conclusions largely stem from the heterogeneous study designs. Studies varied widely in the type of protein consumed, time, dosage and pattern of intake, biomarkers and their measurement methods, exercise type, and participant training status. Future studies should take these factors into consideration when designing studies to address the effects of protein-based interventions on inflammation and oxidative stress. As single studies did show that, in some situations, dietary proteins could modify these pathways, more research is warranted. The potential benefits of such effects are far-reaching; for example, dietary protein supplements are already widely consumed in athletic populations to support skeletal muscle remodeling, but if they can also modify the inflammation/redox status, they could help athletes recover quicker after high-intensity/muscle-damaging exercise, helping them to manage symptoms such as muscle soreness and loss of the force-generating capacity. Moreover, nutritional interventions are currently being explored for their potential to manage low-grade chronic inflammation and oxidative stress, the hallmarks of aging and many diseases. Dietary protein has been recommended as an intervention to manage low-grade chronic inflammation in particular [31], and therefore, high-quality research demonstrating its anti-inflammatory and antioxidant effects is required.

## Figures and Tables

**Table 1:** Studies examining the effects of whole protein supplementation on markers of exercise-induced inflammation and oxidative stress. The Difference column denotes intervention vs. comparator.

Study	Participants	Design	Intervention	Comparator	Timing of Supplement	Exercise	Outcomes	Difference
Baba et al. (2014)	14 M 31 ± 6	Crossover	Drink (22.8-g WPI, 4.4-g CHO)	Drink (5.2 g of the same minus WPI	Pre, during, and post-exercise	$60\;\min\;\mathrm{run}\;(>70\%\;\mathrm{of}\;\dot{V}$O_2 max_)	Plasma IL-6	↔
Betts et al. (2009)	17 M 26 ± 5	SB, Crossover	Drink (0.4-g.kg^−1^ BM·h^−1^ WPI, 1.2-g·kg^−1^ BM·h^−1^ CHO)	Drink (1.2-g·kg^−1^ BM·h^−1^ CHO)	During and ≤4 h post-exercise	90 min LIST	Serum IL-6	↔
							Serum IL-10	↔
							Serum CRP	↔
							Serum IL-1 ra	↔
Buckley et al. (2010)	28 M 24 ± 4	DB, 3-arm Parallel	Drink (1) (25-g WPI) (2) (25-g WPI-HD)	Drink (flavored water)	0, 6 and 22 h post-exercise	100 eccentric contractions of knee extensors	Plasma TNF-α	↔
Draganidis et al. (2017)	11 M 22 ± 1	DB, Crossover	Drink (20-g milk PRO)	Drink (20 g of maltodextrin)	4 × 20 g/day ≤9 h post and 20 g/d for 8 days post-exercise	300 eccentric contractions of knee extensors	Muscle NF-κB	↔
							Muscle HSP70	↔
							Serum PC	↓
							Leukocytes	↔
Grubic et al. (2019)	12 M 22 ± 2	Open label, Crossover	Food bar (20-g WP, 25-g IMO, 7-g fat)	Gel (25-g dextrose)	30-min pre-, mid-way, and post-exercise	RT (3 × 10 reps at 70% of 1 RM) + agility and sprint drills	Serum IFNy	↔
							Serum IL-13	↔
							Serum IL-1 β	↔
							Serum IL-4	↔
							Serum IL-6	↔
							Serum IL-8	↔
							Serum TNF-α	↔
Hall et al. (2013)	10 M 30 ± 8	DB, Crossover	Drink (0.23-g·kg^−1^·h^−1^ casein + 0.1-g·kg^−1^·h^−1^ leucine + 0.87-g·kg^−1^·h^−1^ CHO)	CHO beverage (1.2 g·kg^−1^·h^−1^)	Every 15 min during exercise	2.5-h intermittent cycling (30–90% W_max_)	Neutrophils	↓
							Lymphocytes	↓
Hilkens et al. (2020)	INT: 20 M 24 ± 4 CON: 19 M, 23 ± 4	DB, Parallel	Drink (58.5-g WP + 5.0-g CHO)	Drink (72-g CHO)	2/day for 9 days pre- and post-exercise	10 × 10 DJs with 5 kg vest	Plasma CRP	↔
Karakuş et al. (2020)	22 ± 2 M INT: 15 CON: 9	Parallel	35-g WP drink	NR	3 meals, 3 days post-exercise	RT (10 exercises for different body parts)	Neutrophils	↔
							Platelets	↔
							Leukocytes	↔
							Lymphocytes	↔
Kerasioti et al. (2013)	9 M 28 ± 2	DB, crossover	Cake (0.9-g·kg^−1^·h^−1^ CHO + 0.26-g·kg^−1^·h^−1^ WP)	Isocaloric cake (1.1-g·kg^−1^·h^−1^ CHO + 0.1-g·kg^−1^·h^−1^ WP)	4 × ≤3 h Post-exercise	$2\;h\;\mathrm{cycling}\;\mathrm{at}\;60–65\%\;\dot{V}$$O_{2\max},\;4\;h\;\mathrm{recovery},\;1\;h\;\mathrm{cycling}\;\mathrm{at}\;60–65\%\;\dot{V}$O_2 max_	Plasma IL-6	↓ 4 h post
							Plasma IL-10	↔
							Plasma CRP	↓ 4 h post
Kritikos et al. (2021)	10 M 21 ± 2	DB, 3-arm crossover	Drink (WPI or SOY)—enough to reach 1.5 g/kg/day	Drink (maltodextrin)	1/day for 3 days	60 min speed-endurance training	Glutathione	↔
							Serum TAC	↔
							Plasma PC	↓ SOY vs. PL, 48 h
Naclerio et al. (2014)	10 M 25 ± 4	DB, 3-arm crossover	Drink (53-g CHO,14.5-g WP, 5-g glutamine, 1.5-g L-carnitine-L-tartrate)	Drink 1: (69.5-g CHO) 2: low calorie water	1 dose during exercise and 1 post-exercise	90-min intermittent exercise	Plasma IL-6	↔
Nieman et al. (2020)	Pea: 31 M 37 ± 2 WP: 31 M 40 ± 2 CON: 30 M 38 ± 2	DB, 3-arm parallel	Drink (1) Pea PRO (0.3 g·kg^−1^) (2) WPI (0.3 g·kg^−1^)	Drink (water)	13 doses over 5 days on day of and post-exercise	90-min eccentric exercise	Serum CRP	↔
Rankin et al. (2017)	10 F 22 ± 2	Crossover	Drink (17-g milk PRO; 25.5-g CHO)	Drink (52.6-g CHO)	<30 min post-exercise	Intermittent sprint cycling (~60 min)	Serum hsCRP	↔
							Serum GSH/GSSG	↔
							Serum PC	↔
Rothschild et al. (2021)	17 M 31 ± 12	Crossover	PRO-rich meal (0.45-g·kg^−1^ WPI + 0.24-g·kg^−1^ fat)	CHO-rich meal (1-g·kg^−1^ CHO)	30 pre-exercises	HI cycling (~45 min)	Urinary F2-Isoprostanes	↔
Rowlands et al. (2016)	12 M cyclists 30 ± 7	SB, 3-arm crossover	Drink 1: WP (70 g, 15-g LEU, 180-g CHO, 30-g FAT) 2: WP (23.5 g, 5-g LEU, 180-g CHO, 30-g FAT)	Isocaloric drink (0-g WP, 274-g CHO, 30-g FAT).	≤90 min post-exercise (in 4 doses)	HI cycling (70–90% W_max_,100 min)	* Muscle inflammatory-myogenic regenerative processes	↑
Shenoy et al. (2016)	40 M 20 ± 2	DB, Parallel	Drink (21-g SOY, 21-mg isoflavones)	Drink (sweetened water)	2/day for 4 weeks	100 DJs	Plasma hsCRP	↔
							Plasma MPO	↔
Wells et al. (2017)	10 M 25 ± 3	Crossover	Drink (Milk PRO 20-g AAs, 6-g CHO)	Drink (Flavored water, 2.5-g CHO)	Post-exercise	Lower body RT (10–12 reps at 70% 1 RM)	Plasma TNF-α	↔
							TNFr1 expression on monocytes	↔
Wojcik et al. (2001)	27 M 24 ± 1	3-arm parallel	Drink (1) Milk (0.9-g·kg^−1^ CHO, 0.4-g·kg^−1^ PRO) (2) CHO (1.25 g·kg^−1^)	Drink (sweetened water)	2 × post-exercise	100 eccentric contractions of knee flexors	Serum IL-6	↔
							Serum IL-1	↔
							Serum TNF	↔

Data presented as mean ± SD. M = male; F = female; PL = placebo; CHO = carbohydrate; PRO = protein; WP = whey protein; WPI = whey protein isolate; SB = single-blinded, DB = double-blinded, BM = body mass; INT = intervention; CON = control; IL-6 = interleukin 6; IL-10 = interleukin 10; IL-1 = interleukin-1; IL-1 ra = interleukin-1 receptor antagonist; CRP = C-reactive protein; hsCRP = high sensitivity C-reactive protein IMO = isomalto-oligosaccharides; TNF = tumor necrosis factor; TNF-α = tumor necrosis factor-alpha; TNFr1 = expression of tumor necrosis factor receptor 1; MPO = myeloperoxidase; AA = amino acid; LEU = leucine; SOY = soy protein; TAC = total antioxidant capacity; NF-κB = nuclear factor kappa-B; HSP70 = 70 kilodalton heat shock proteins; NR = not reported; RT = resistance training; 1 RM = one-repetition maximum; reps = repetitions; IFNy = interferon gamma; IL-13 = interleukin 13; IL-1ß = interleukin-1-beta; IL-4 = interleukin 4; IL-8 = interleukin 8; PC = protein carbonyls; DJs = drop jumps; GSH/GSSG = reduced glutathione to oxidized glutathione ratio; HI = high intensity; LIST= Loughborough intermittent shuttle test; ↔ = no significant difference; ↓= significantly decreased responses; ↑ = significantly increased responses. * This study measured several hundreds of makers in muscle tissue, and thus, the general conclusion from the data is presented here instead.

**Table 2:** Studies examining the effects of various amino acids on the markers of exercise-induced inflammation and oxidative stress. The Difference column denotes intervention vs. comparator.

Study	Participants	Design	Intervention	Comparator	Timing of supplement	Exercise	**Outcomes**	**Difference**
**Mixed Amino Acids**								
Jackman et al. (2010)	INT: 12 M CON:12 M	SB, Parallel	Drink (BCAA: 3.5-g LEU, 2 g of isoleucine, + 1.7 g of valine)	Drink (sweetened water)	4/day on the day of and 3 days post-exercise	120 eccentric knee extensions	Serum IL-6	↔
Ra et al. (2013)	36 M 9 per group 22 ± 4	DB, 4-arm parallel	Drink: 1: BCAA (3.2 g) + taurine (2 g) 2: BCAA (3.2 g) + PL 3: Taurine (2 g) + PL	Starch (to match treatment volumes)	3/day pre- and post- exercise for 18 days	Eccentric elbow flexor exercises (6 × 5 reps at 90% MVC)	Serum 8-OHdG	↓ BCAA + taurine vs. BCAA + PL and PL
Takegaki et al. (2020)	INT: 10 M 21 ± 1 CON:10 M 22 ± 2	DB, parallel	Drink (5-g Leucine-enriched Aas)	Drink (water)	2.5 g × 2, pre- and post-exercise	Lower body RT (3 × 10 reps at 70% of 1 RM)	IL-6 muscle mRNA	↔
							IL-1β muscle mRNA	↔
Waskiw-Ford et al. (2020)	INT: 10 M 24 ± 4 CON:10 M 23 ± 5	DB, parallel	Drink (4 g of essential Aas)	Isocaloric CHO PL	3/day, for 4 days post-exercise	Lower-body RT (5 × 9–12 reps at 75% of 1 RM)	Muscle HSP25	↔
							Plasma IL-6	↔
							Muscle HSP72	↓
Wells et al. (2016)	10 M 25 ± 3	Crossover	Drink (20 g of milk PRO, 6 g of CHO)	Drink (flavored water, 2.5-g CHO)	Post-exercise	Lower-body RT (6 × 10–12 reps at 70% of 1 RM)	Plasma MCP-1	↔
							CCR2	↔
							CD11 b	↔
							CD14^+^ MON	↔
							CD14 ^++^ CD16^-^ MON	↔
**β-hydroxy-β-methylbutyrate**								
Wilson et al. (2013)	INT: 11 M 20 ± 5 CON: 9 M 22 ± 2	Parallel	Drink (3-g·day^−1^ HMB-FA)	Drink (sweetened water)	Pre- and post-exercise	Full body RT (3 × 12 reps)	Plasma CRP	↔
**L-carnitine**								
Parandak et al. (2014)	INT: 10 M 22 ± 3 CON:11 M 22 ± 3	DB, Parallel	Capsules (2-g L-carnitine)	Capsules (2-g lactose)	Daily for 2-wk pre-exercise	$14-\mathrm{km}\;\mathrm{run}\;\mathrm{at}\;50\%\;\dot{V}$O_2 max_	Serum TAC	↑ 24 h post
							Serum TBARS	↓ 24 h post
Volek et al. (2002)	10 M 24 ± 2	Crossover	Capsules (2-g·day^−1^ L-carnitine 944-mg·day^−1^ L-tartrate)	Capsules (cellulose)	Daily for 3-wk, pre and post-exercise	Lower body RT (5 × 15–20 reps at 50% of 1 RM)	Plasma MDA	↓ at 15 min post
							Plasma XO	↓ at 0, 15, 180 min post
**Citrulline**								
Sureda et al. (2009)	22 ± 4 INT: 8 M CON: 9 M	DB, Parallel	Drink (6-g citrulline-malate)	Drink (lemon juice)	2 h pre-exercise	137.1 km cycling	PMN-ROS	↑ post
							PMN-MDA	↔
							DNA damage	↔
**Collagen peptides**								
Clifford et al. (2019)	INT: 12 M 24 ± 4 CON: 12 M 25 ± 5	DB, Parallel	Drink (20-g·day^−1^ CP)	Drink (20-g maltodextrin)	10 g × 2, 7 days pre and 2 days post-exercise	150 DJs	Leukocytes	↔
							Neutrophils	↔
							Monocytes	↔
							Lymphocytes	↔
							Serum IL-6	↔
**Glutamine**								
Cury-Boaventura et al. (2008)	9 M 25 ± 4	DB, Crossover	Drink (2.8-g WP,+ 175-mg glutamine, 50-g maltodextrin)	Drink (50-g maltodextrin)	30 min pre-exercise	Treadmill running to exhaustion	DNA damage in leukocytes	↔
							Neutrophil O_2_^−^	↔
Nakhostin-Roohi et al. (2017)	INT: 9 M 25 ± 1 CON:10 M 22 ± 1	DB, Parallel	Drink (1.5-g·kg^−1^ BM·day^−1^ glutamine)	Drink (sweetened water)	1/day for 7 days pre-exercise	$14-\mathrm{km}\;\mathrm{run}\;\mathrm{at}\;50\%\;\mathrm{of}\;\dot{V}$O_2 max_	Plasma TAC	↔
							plasma Glutathione	↔
							Plasma MDA	↔
Nemati et al. (2019)	INT: 15 M 19.7 ± 2 CON: 15 M 19 ± 1	Parallel	Drink (0.3-g·kg^−1^ BM·day^−1^ glutamine + 25-g sugar)	Drink (25-g sugar)	1/day for 14 days pre-exercise	$\dot{V}$O_2 max_	Serum TAC	↑
							Serum MDA	↓
							Serum hsCRP	↓
							Glutathione	↑
**Taurine**								
Da Silva et al. (2014)	21 ± 6 INT: 11 M CON: 10 M	DB, Parallel	Capsules (Taurine 50-mg·kg BM^−1^·day^−1^)	Capsules (Starch 50-mg·kg BM^−1^·day^−1^)	1/day, 14 days pre and 7 days post-exercise	Eccentric elbow flexion and extension exercise (3 × 11–15 reps at 80% of 1 RM)	Xylenol	↓
							Plasma PC	↓
							Plasma TT	↑
							Erythrocyte-derived SOD	↔
							Erythrocyte-derived CAT	↔
							Erythrocyte-derived GPX	↔
							Plasma TNF-α	↔
							Plasma IL-1ß	↔
							Plasma IL-10	↔
Ra et al. (2016)	INT: 15 M 25 ± 1 CON: 14 M 25 ± 1	DB, Parallel	Powder (6-g taurine)	Powder (6-g lactose)	Daily for 14 days pre-, on, & 3 days post-exercise	Eccentric contractions of the elbow flexors (2 × 20 reps)	Serum MDA	↓ 3 d, 4 d post
Zembron-Lacny et al. (2007)	INT: TAU: 15 M, 22 ± 1 CON: (1) PL: 15 M 22 ± 1 (2) NAC: 15 M, 22 ± 2 (3) LIP: 10 M, 23 ± 1	4-arm parallel	Drink TAU (3 g·day^−1^)	Drink (1) PL (0.35-g·day^−1^ saccharum lactis) (2) NAC (1.8 g·day^−1^) (3) LIP (1.2 g·day^−1^)	3/day for 3 days pre-exercise	Full body RT (3 exercises in a circuit until exhaustion)	SOD	↑ TAU vs. PL 24 h post
							GPX	↑ TAU vs. PL 24 h post
							CAT	↔
							Plasma Protein thiols	↔
							TBARS	↔

Data presented as mean ± SD. INT = intervention; CON = control; PL = placebo; M = male; F = female; SB = single-blinded, DB = double-blinded, BM = body mass; CHO = carbohydrate; PRO = protein; IL-6 = interleukin 6; MVC = maximal voluntary contraction; IL-1β = interleukin-1-beta; 1 RM = one-repetition maximum; reps = repetitions; HSP25 = heat shock proteins 25; HSP72 = heat shock protein 72; TNF-α = tumor necrosis factor-alpha; IL-10 = interleukin 10; HMB-FA = β-hydroxy-β-methylbutyrate free acid; BCAA = branched-chain amino acid; CAT = catalase; GPX = glutathione peroxidase; LEU = leucine; LIP, α-lipoic acid; MCP-1 = monocyte chemoattractant protein 1; CCR2 = chemokine receptor type 2; CRP = C-reactive protein; hsCRP = high sensitivity C-reactive protein; NAC = N-acetylcysteine; PC = protein carbonyls; PMNs = polymorphonuclear neutrophils; MDA = malondialdehyde; TAC = total antioxidant capacity; TAU = taurine; LIP = α-lipoic acid; DJs, drop jumps; ROS = reactive oxygen sepsis; RT = resistance training; SOY = soy protein; SOD = superoxide dismutase; TBARS = thiobarbituric acid-reactive substance; XO = xanthine oxidase; TT = total thiol content; Neutrophil O_2_^−^ = superoxide production in neutrophils; CP = collagen peptides; 8-OHdG, 8-hydroxydeoxyguanosine; CD11 b = expression of macrophage-1 antigen in cluster of differentiation molecule 11 b; CD14^+^ MON = percent change in proportion of CD14^+^ monocytes relative to all leukocytes; CD14^++^CD16^-^ MON = percent change in classical monocytes relative to all monocytes; ↔ = no significant difference; ↓ = significantly decreased responses; ↑ = significantly increased responses.

## Data Availability

Data can be provided at reasonable request from the corresponding author.

## Supplementary Materials

The following are available online at https://www.mdpi.com/article/10.3390/antiox11010013/s1: Search strategy: Figure S1: Risk of bias summary for individual studies with a parallel design. Figure S2: Risk of bias summary for individual studies with a crossover design.
