# Supplementary material for: The Effects of Dietary Protein Supplementation on Exercise-Induced Inflammation and Oxidative Stress: A Systematic Review of Human Trials
Source: Antioxidants (Basel). 2021 Dec 22;11(1):13. doi: 10.3390/antiox11010013 (PMC8773319; doi:10.3390/antiox11010013)
Supplement: Supplementary file 1 [file antioxidants-11-00013-s001.zip › antioxidants-1515183-supplementary.pdf]

Online Supplementary Material

Search strategy

	Search terms	**Ebsco: Medline**	**SPORTDiscuss**
S1	SU (protein* or "protein supplementation") OR TI (protein* or "protein supplementation") OR AB (protein* or "protein supplementation")	4,593,558	32,989
S2	SU (“whey protein*” or "casein protein*" or “plant protein*” or “milk”) OR TI (“whey protein*” or "casein protein*" or “plant protein*” or “milk”) OR AB (“whey protein*” or "casein protein*" or “plant protein*” or “milk”)	234,217	5,503
S3	SU (“Leucine*” or "BCAA” or “branch chain N3 acid*" or “hydroxymethylbutyrate” or “beta-hydroxy beta-methylbutyric acid” or “HMB*” or “taurine” or “collagen*” or “gelatin*” or “L-glutamine” or “glutamine” or “amino acid*” or “L-arginine” or “L-citrulline”) OR TI (“Leucine*” or "BCAA” or “branch chain N3 acid*" or “hydroxymethylbutyrate” or “beta-hydroxy beta-methylbutyric acid” or “HMB*” or “taurine” or “collagen*” or “gelatin*” or “L-glutamine” or “glutamine” or “amino acid*” or “L-arginine” or “L-citrulline”) OR AB (“Leucine*” or "BCAA” or “branch chain N3 acid*" or “hydroxymethylbutyrate” or “beta-hydroxy beta-methylbutyric acid” or “HMB*” or “taurine” or “collagen*” or “gelatin*” or “L-glutamine” or “glutamine” or “amino acid*” or “L-arginine” or “L-citrulline”)	1,338,713	10,570
S4	S1 OR S2 OR S3	5,243,237	43,113
S5	(MH "Exercis*") OR (MH "Exercise test*")<br>(DE "EXERCISE") OR (DE "EXERCISE tests")	214,352	96,588
S6	TX (eccentric N3 exercise) or (endurance N3 exercise) or (repeated sprint* N3 exercise) or (sprint* N3 exercise) or (resistance N3 exercise) or (strength N3 exercise) or (aerobic N3 exercise) or (anaerobic N3 exercise)	33,323	29,076
S7	TX (exercise and (musc* N2 damage*)) OR (exercise and (musc* N2 injur*)) OR (exercise and (musc* soreness))	6,076	9,054

S8	TX (exercise induced musc* damage) or (eccentrically induced musc* damage*)	1,153	2,298
S9	TX EIMD	209	272
S10	TX (delayed onset N2 muscle soreness) or DOMS	10,891	3,170
S11	TX (musc* N3 recovery) OR (musc* N3 repair)	8,779	6,374
S12	TX (musc* N3 sore*) or (musc* N3 pain*) or (musc* N3 damag*)	33,330	12,950
S13	TX (exercise and ((Skeletal N2 damage*)) or (exercise and (Skeletal N2 injur*)) or (exercise and (Skeletal N2 soreness))	555	413
S14	S5 OR S6 OR S7 OR S8 OR S9 OR S10 OR S11 OR S12 OR S13	276,083	131,634
S15	S4 AND S14	22,379	6,246
S16	MH "Inflammation"<br>DE "INFLAMMATION"	170,964	 3,209
S17	TX (inflammat* N3 marker*) OR (inflammat* N3 response*) OR (inflammat* N3 process*) OR (anti-inflammat* N3 marker*) OR (inflammation)	700,863	13,696
S18	TX (marker* N3 musc* damage) OR (marker* N3 musc* sore*) OR (marker* N3 musc* inflammat*) OR (marker* N3 musc* injur*)	1,278	1,527
S19	MH (Oxidative Stress) OR (Oxidative Damage)<br>DE (Oxidative Stress) OR (Oxidative Damage)	143,670	 3,523
S20	TX (marker* N3 oxidative stress)	15,200	1,926
S21	TX (redox)	195,711	3,588
S22	TX (reactive N3 oxygen species)	170,394	5,999
S23	TX (free radicals)	118,725	6,442
S24	S16 OR S17 OR S18 OR S19 OR S20 OR S21 OR S22 OR S23	1,138,743	25,095
S25	S15 AND S24	4,878	1,474
S26	TX (randomized controlled trial*) OR (RCT*)	802,276	44,726
S27	TX (randomi?ed control* trial*) OR TX ("randomi?ed N3 trial*" or "randomi?ed W3 trial*") OR TX (randomly) OR TX (trial*) OR TX ("placebo controlled" or "placebo N3 trial*)	2,153,763	213,634
S28	TX ((clinic* N3 trial) or (controlled N3 trial) or (comparative N3 trial) or (placebo N3 trial) or (prospective N3 trial)) or TX ((clinic* N3 study) or (controlled N3 study) or (comparative N3 study) or	4,132,308	187,786

	(placebo N3 study) or (prospective N3 study) or (randomi?ed N3 study))		
S29	S26 OR S27 OR S28	4,598,415	296,014
S30	S25 AND S29	1,693	857
S31	S30 NOT AB (mice or rats or rodents or animal) NOT SU (mice or rats or rodents or animal) NOT TI (mice or rats or rodents or animal)	1,372	693

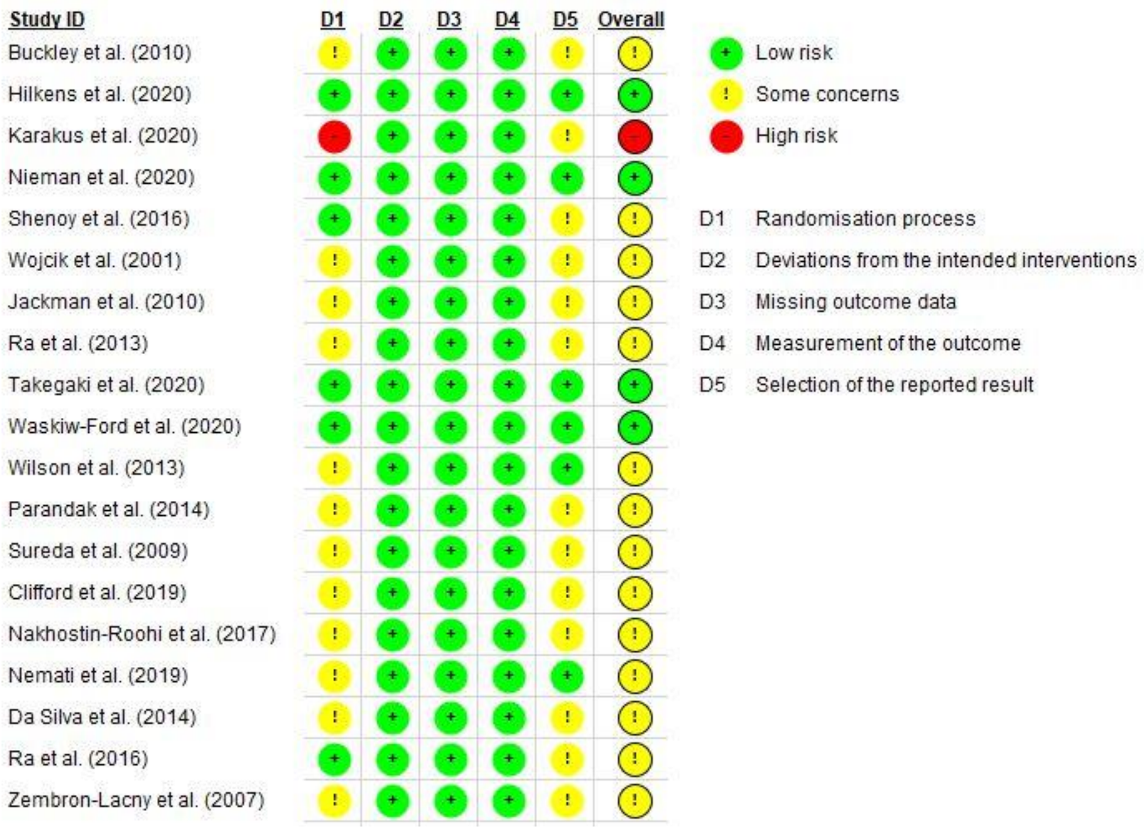

Figure S1: Risk of bias summary for individual studies with a parallel design.

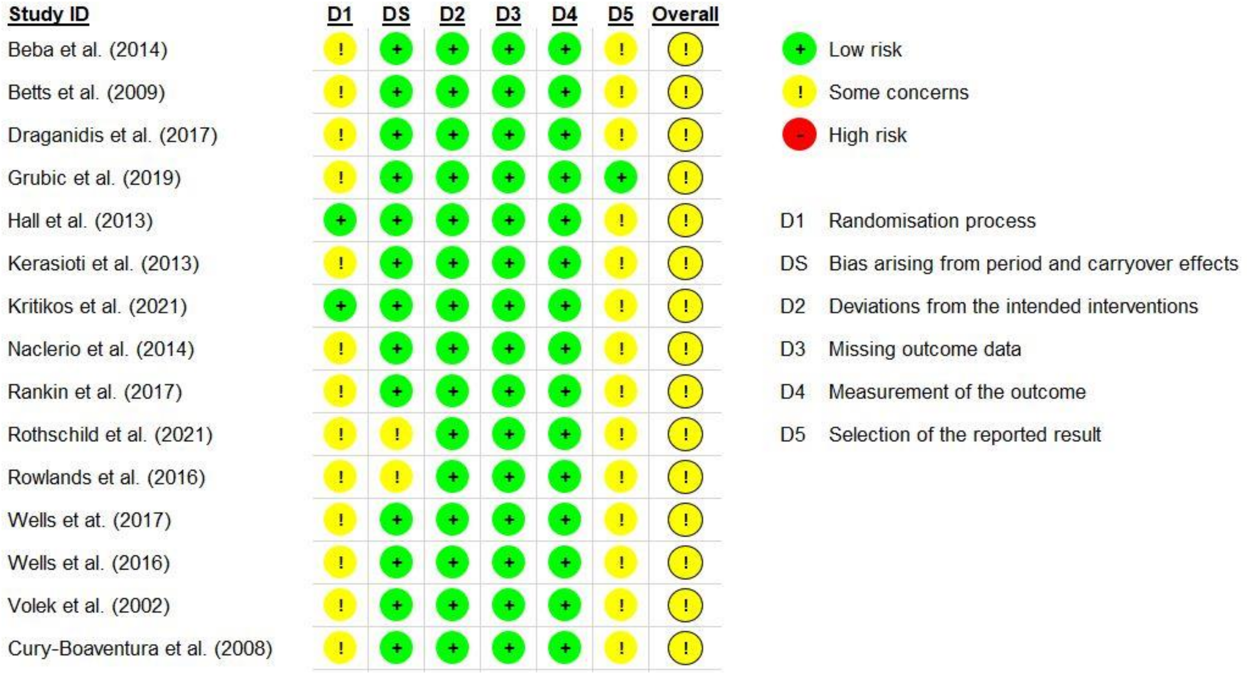

Figure S2: Risk of bias summary for individual studies with a crossover design.
